# Prevalence and Antibiotic Resistance Pattern of Methicillin-Resistant *Staphylococcus aureus* from an Orthopaedic Hospital in Nigeria

**DOI:** 10.1155/2013/860467

**Published:** 2013-10-27

**Authors:** C. E. Udobi, A. F. Obajuluwa, J. A. Onaolapo

**Affiliations:** ^1^Department of Pharmaceutics and Pharmaceutical Technology, University of Uyo, Nigeria; ^2^Department of Pharmaceutics and Pharmaceutical Microbiology, Ahmadu Bello University, Zaria, Nigeria

## Abstract

Patients with surgical wounds have been reported to be at high risk of MRSA carriage and infection. The prevalence and antibiotic resistance pattern of this organism in the orthopaedic ward of Ahmadu Bello University Teaching Hospital (ABUTH), Zaria-Nigeria, a 547-bed Nigerian hospital, were thus studied. A total of 185 isolates of *Staphylococcus aureus* were confirmed from 217 samples taken from the orthopaedic wards of the hospital using standard isolation methods. Out of these, 44 (23.8%) were from the wounds of patients and 70 (37.8%) from the skin. The remaining 65 (35.1%) and 6 (3.2%) were from their beds and the atmospheric air, respectively. Out of these, 33 (75%), 36 (51.4%), and 48 (73.8%) from wounds, skin, and bed, respectively, were found to be methicillin-resistant *Staphylococcus aureus* (MRSA) using the disc-sensitive test methods. None was detected from the atmosphere. The antibiotic susceptibility pattern results showed the level of resistance to be ampicillin 100% in all the three sites, pefloxacin 90.9%, 72.2%, 66.7%, ceftriaxone 69.7%, 72.2%, 70.8%, gentamicin 54.5%, 52.8%, 37.5%, and ciprofloxacin 51.5%, 47.2%, 35.4% at the wound, skin, and bed sites, respectively. Results confirm that MRSA continues to pose a threat to the hospitalized patients, especially those with bone and wound infections.

## 1. Introduction

Methicillin-resistant *Staphylococcus aureus* (MRSA) are isolates of *Staphylococcus aureus* which have acquired genes encoding antibiotic resistance to all penicillins including methicillin. This resistance is mediated by an altered penicillin binding protein (PBP2a) which is encoded by the Mec A gene [1, 2]. They were first discovered in the United Kingdom in 1961 [3] but have now become a major clinical problem worldwide [4]. *Staphylococcus aureus* generally has been implicated in bone and wound infections encountered in orthopaedic practice, for example, osteomyelitis, as well as in postoperative wound infections where they are known to lead to delayed healing of wounds, delayed union, or even nonunion of bones which may lead to the amputation of such bones.

Numerous studies have shown that *Staphylococcus aureus* is among the most frequently encountered microorganisms in microbiology laboratories in Nigeria [5–7]. However, information on the prevalence of MRSA in Nigeria is insufficient despite the established fact that MRSA is a significant health threat. Precisely patients with surgical wounds have been reported to be at high risk of MRSA infection [8].

In the Orthopaedics Department of Ahmadu Bello University Teaching Hospital (ABUTH), the commonly prescribed antibiotics are gentamicin, ciprofloxacin, and ampiclox. Even though there has not been a reported case of MRSA epidemic in this department, this study has become very expedient because of the significant epidemic potential of these organisms and the high morbidity and mortality rates they cause in humans with rapid development of resistance which has made it into a major clinical problem worldwide.

This report presents the results of the study of the prevalence of methicillin-resistant *Staphylococcus aureus* in orthopaedic patients/wards in Ahmadu Bello University Teaching Hospital Zaria, Nigeria, and the susceptibility pattern of these isolates to a wide range of antibiotics especially those commonly prescribed in the orthopaedic department of the hospital. Methicillin-resistant *Staphylococcus aureus* is known to have a significant epidemic potential. This report will therefore serve as a guide in handling reported cases of MRSA in orthopaedic patients, especially as it is concerned with the empirical selection of antimicrobial therapy and the need to pay close attention to cleanliness and other measures to prevent the spread of MRSA strains among patients and other hospital staff.

## 2. Materials and Methods

### 2.1. Isolation and Purification of *Staphylococcus aureus*

All staphylococci used were isolated from the wounds, beds, and skin of orthopaedic patients in the orthopaedic ward of Ahmadu Bello University Teaching Hospital Zaria, Nigeria, a 547-bed specialist and teaching hospital, using sterile cotton swabs moistened in sterile peptone water. The swab was firmly applied, slowly rotated thoroughly covering the surface of the wound and/or fractured area. The same was done for patient beddings and skin (the skin samples were taken from the exposed parts of the body where there were no wounds of any sort). The swab was then dropped in a sterile nutrient broth, placed in an ice pack, and taken to the laboratory to be incubated at 37°C for 18 hours. Observed colonies were further screened for methicillin resistance ability and stored in the refrigerator at 4°C on agar slants.

### 2.2. Identification of Methicillin Resistance *Staphylococcus aureus*

All positive agar slats from the above were further screened for their methicillin resistance capability. This was done according to the NCCLS (2002) guidelines using oxacillin in agar screen test. Isolates from a solution adjusted to 0.5 McFarland standard were spot inoculated unto Mueller Hinton agar supplemented with 6 *μ*g/mL oxacillin and 4% sodium chloride. The plates were incubated at 35°C for 24 hours. The isolates that survived showing more than one colony were considered methicillin resistant.

### 2.3. Antibiotic Susceptibility Test

The Clinical Laboratory Standard Institute (CLSI) modified disc agar diffusion technique was used. Discrete colonies of confirmed MRSA isolates growing on nutrient agar plates were emulsified in 3 mL of phosphate buffered solution (PBS) and the turbidity was adjusted to 0.5 McFarland standard. Using a sterile swab stick, the surface of Mueller Hinton agar in a 90 mm diameter plate was inoculated with the bacterial suspension by streaking the surface of the agar in three directions, rotating the plate approximately 60° to ensure even distribution. The plates were allowed to dry for 10 minutes before antibiotic discs were aseptically applied to the surface of the agar. They were allowed a further drying period of 30 minutes and then incubated at 35°C. Similar treatment was given to *Staphylococcus aureus* ATCC25923 which was used as a positive control. The diameter of zones of inhibition produced by each antibiotic disc was measured and the isolates were classified as resistant, intermediate, and sensitive based on the standard interpretative chart of the NCCLS and Fluka zone interpretative chart in accordance with WHO requirement.

### 2.4. Determination of Multiple Antibiotic Resistance (MAR) Index

The MAR index was determined for each isolate by dividing the number of antibiotics to which the organism is resistant by the total number of antibiotics tested [9, 10]. MAR index gives an indirect suggestion of the probable source(s) of an organism.

## 3. Results

From a total of 217 samples obtained from the hospital, 185 isolates of *Staphylococcus aureus* (85.25%) were confirmed. 44 (23.8%) of these were from the wounds of patients and 70 (37.8%) were from their skins, while 65 (35.1%) and 6 (3.2%) were from their beds and the atmosphere of the hospital.

The diameter of the zones of inhibition of the isolates to various antibiotics was interpreted using the standard interpretative chart updated according to the current Clinical Laboratory Standard Institute (CLSI) standard and Fluka zone interpretative chart, in accordance with WHO requirements. The results are shown on Table 1. The percentage of susceptibility of *Staphylococcus aureus* isolates from different sites to different antibiotics is shown on Table 2.

### 3.1. Detection of Methicillin Resistance

Results obtained showed that 33 (75%) of the 44 staphylococcal isolates from wounds screened were methicillin resistant. Out of the 70 skin isolates screened, 36 (51.4%) were methicillin resistant and 48 (73.8%) out of the 65 bed isolates were also methicillin resistant.

### 3.2. Resistance Pattern of MRSA to Antibiotics

Table 1 shows the resistance pattern of MRSA isolates from various sites against a number of antibiotics. The results show that the pattern is in the following (descending) order: ampicillin > pefloxacin > ceftriaxone > gentamicin > ciprofloxacin.

## 4. Discussion

Results obtained from this study show that the prevalence of MRSA isolates (wound (75%), skin (51.4%), and bed (73.85)) ( all from in-patients) obtained is reasonably high. Previously, Ikeh [11] had reported an MRSA prevalence of 43.5% in Jos, Nigeria, of which 81% was from in-patients. Nwakwo [12], reported 28.6% prevalence in kano Nigeria, with 62% of these from in-patients. Taiwo et al., 2004 [13], had earlier reported a prevalence of 34.7 in the city of Ilorin with 70.6% of this being from in-patients.

The high incidence of MRSA infection in the hospital studied may not be unconnected with the location of the hospital. The hospital is located in the far northern part of Nigeria where enlightenment status of the people may not be as high as it is in Lagos and Ibadan (Southern part of Nigeria) that formed part of the study of Shittu et al. (2012) [14]. If this factor is considered, the relatively lower incidences observed in the other results from the other parts of the country can be explained.

The high prevalence observed in this study which is also an in-patient, study (all the samples were taken from in-patients) and those of the in-patients in the other studies reported, indicate that MRSA continues to be a menace in Nigerian hospitals and that the spread is no doubt hospital-aided. This may not be unconnected therefore with the hospital environment, for example, arrangement of people in rooms and wards which makes transfer of these organisms among in-patients easier. Poor hygienic conditions and nonadherence to or even the lack of a relevant antibiotic policy have been suggested as possible reasons for these high carriage rates [3]. These suggestions continue to remain relevant.

The detection of the Mec A gene remains the golden standard and the most acceptable method of MRSA identification even though other methods like the NCCL 2002 method employed in this investigation are reported to be highly specific in identifying MRSA [15]. This comparatively higher prevalence percentage obtained from a part of Nigeria, where no reasonable report of MRSA prevalence has been made before now, is certainly a matter of concern. Results obtained showed the highest resistance to ampicillin (100% for all The isolates) and the highest susceptibility to ciprofloxacin (56.3% for bed isolates) (Table 1). The very high resistance to ampicillin, a penicillin, is understandable since all MRSA strains have been variously reported to be resistant to all *β*-lactam antibiotics of which ampicillin is one [16, 17].

Analysis of the results of multiple antibiotic resistances (MAR) index determined for each isolate by dividing the number of organisms to which the organism is resistant by the number of antibiotics tested showed that 79.6%, 60.6%, and 76.5% of wound, skin, and bed isolates have MAR index greater than 0.25% (Table 2). This suggests that the isolates originated from a high risk source of contamination where antibiotics are often used and possibly abused.

The greatest problem with the control of resistant organisms in the Northern part of Nigeria has remained that of education. Very high indiscriminate use of antibiotics (without prescription) is common knowledge. This explains why the MAR index is high pointing to an external source of contamination outside the hospital. Education and more education especially of the local populace remain the most important step to halting a rise in this infection.

## 5. Summary

The prevalence rate of MRSA observed in the study is high. MAR index results obtained show a possibility of misuse and abuse of antibiotics among the populace even before they come to hospital. The highest susceptibility was obtained with ciprofloxacin among the antibiotics used.

## 6. Recommendation

Given the high prevalence obtained in this investigation, it will be necessary for the government and medical personnel, especially those involved in the daily prescription of these commonly used antibiotics, to pay greater attention to the situation. It is not impossible that these patients may have encountered these organisms before coming to the hospital; therefore, continuous education on cleanliness and the dangers of the misuse of antibiotics cannot be overemphasized.

## Figures and Tables

**Table 1:** Resistance pattern of MRSA isolates from various sites (wound, skin, and bed) to antibiotics.

Antibiotics	Wounds			Skin			Bed		
	No. of isolates (%)			No. of isolates (%)			No. of isolates (%)		
	R	I	S	R	I	S	R	I	S
AMP_10_	33 (100)	None	None	36 (100)	None	None	48 (100)	None	None
CN_10_	18 (54.5)	7 (21.2)	8 (24.2)	19 (52.8)	5 (13.9)	12 (33.3)	18 (37.5)	8 (16.7)	22 (45.8)
CRO_30_	23 (69.7)	7 (21.2)	3 (9.1)	26 (72.2)	6 (16.7)	4 (11.1)	34 (11.1)	6 (12.5)	5 (10.4)
PEF_5_	30 (90.9)	None	3 (9.1)	26 (72.2)	3 (8.3)	7 (19.4)	32 (66.7)	3 (6.3)	13 (27.1)
CIP_5_	17 (51.5)	3 (9.1)	13 (39.4)	17 (47.2)	3 (47.2)	16 (44.4)	17 (35.4)	4 (8.3)	27 (56.3)

R: resistance, I: intermediate, S: sensitive, Amp_10_: ampicillin 10 *µ*g, CN_10_: gentamicin 10 *µ*g, CRO_30_: ceftriaxone 30 *µ*g, PEF_5_: pefloxacin 5 *µ*g, CIP_5_: ciprofloxacin 5 *µ*g.

**Table 2:** Multiple antibiotic resistance index of wound, skin, and bed isolates.

MAR index	Number of isolates (%)		
	Wound	Skin	Bed
0.1	8 (18.2)	19 (28.8)	7 (10.9)
0.2	None	None	None
0.3	1 (2.3)	7 (10.6)	8 (12.5)
0.4	3 (6.8)	3 (4.5)	5 (7.8)
0.5	5 (11.4)	4 (6.1)	10 (15.6)
0.6	4 (9.1)	9 (13.6)	8 (12.5)
0.7	None	None	None
0.8	7 (15.9)	6 (9.1)	9 (14.1)
0.9	7 (15.9)	11 (16.7)	7 (10.9)
1.0	9 (20.5)	7 (10.6)	10 (15.6)

MAR values greater than 0.25 are considered to represent exposure to point source contamination [9].
